# Use of fecal microbiome to understand the impact of housing conditions on metabolic stress responses in farmed saltwater crocodiles (*Crocodylus porosus*)

**DOI:** 10.3389/fvets.2025.1496946

**Published:** 2025-02-13

**Authors:** David J. Beale, Thao V. Nguyen, Tim Dyall, Jodie van de Kamp, Andrew Bissett, Leisha Hewitt, Alison H. Small

**Affiliations:** ^1^Environment, Commonwealth Scientific and Industrial Research Organisation (CSIRO), Ecosciences Precinct, Dutton Park, QLD, Australia; ^2^Agriculture & Food, Commonwealth Scientific and Industrial Research Organisation (CSIRO), Armidale, NSW, Australia; ^3^Environment, Commonwealth Scientific and Industrial Research Organisation (CSIRO), Battery Point, TAS, Australia; ^4^Roseworthy Campus, School of Animal and Veterinary Sciences, The University of Adelaide, Roseworthy, Australia

**Keywords:** metabolomics, bacterial community sequencing, corticosterone, animal welfare, captivity, reptiles

## Abstract

**Introduction:**

Understanding the impact of housing conditions on the stress responses in farmed saltwater crocodiles (*Crocodylus porosus*) is crucial for optimizing welfare and management practices.

**Methods:**

This study employed a multi-omics methodology, combining targeted and untargeted LC–MS for metabolite, lipid, and hormone profiling with 16S rRNA gene sequencing for microbiome analysis, to compare stress responses and changes in fecal samples of crocodiles housed in single versus group pens. Metabolic responses to a startle test were evaluated through multivariate analysis, and changes post-stress were examined.

**Results:**

A total of 564 metabolic features were identified. Of these, 15 metabolites were linked to the cortisol biosynthesis pathway. Metabolite origin analysis showed that 128 metabolites originated from the host, 151 from the microbiota, and 400 remained unmatched. No significant differences in fecal corticosterone levels were observed between single and group pens. However, metabolic profiling revealed distinct differences in stress responses: single pen crocodiles exhibited downregulation of certain compounds and upregulation of others, affecting pyrimidine and purine metabolism pathways when compared to grouped pen crocodiles, linked to altering energy associated induced stress. Additionally, fecal microbiome analysis indicated increased Firmicutes:Bacteroides (F:B) ratio in group-housed animals, suggesting greater stress.

**Discussion:**

The study highlights that while traditional stress indicators like corticosterone levels may not differ significantly between housing conditions, metabolic and microbiome analyses provide deeper insights into stress responses. Single pens are associated with less metabolic disruption and potentially better health outcomes compared to group pens. These findings underscore the value of fecal microbiome and metabolomics in assessing animal welfare in farmed crocodiles.

## 1 Introduction

The demand for luxury products made from crocodile leather has driven the global expansion of crocodile farms. Over 11 different species of crocodilians are farmed worldwide for their meat and skin products (1). Saltwater or estuarine crocodiles (*Crocodylus porosus*) are particularly prized for their superior skins, attributed to the absence of bony deposits (osteoderms) in their ventral scales, resulting in a higher number of small, evenly distributed scales (2–4). Key producers of *C. porosus* include Australia, Bangladesh, Indonesia, Malaysia, Papua New Guinea, the Philippines, and Thailand (1). In Australia, crocodile farming began in the 1970s, although a sustainable industry did not emerge until the 1980s (5, 6). These farms are primarily located in tropical northern regions, focusing on exporting skins and providing other by-products like meat, feet, teeth, and skulls to the domestic market (6). The value of skins accounts for 80% of the total product value, with meat and other by-products contributing 15 and 5%, respectively (7).

Crocodiles can be reared in group or individual pens (6). Single pens offer benefits like easier monitoring, less social stress, better health, and improved growth and skin quality (6). However, they are costlier to construct and manage (6). Group pens are cheaper, but come with management challenges and increased social conflict, leading to health issues and lower skin product quality (6). Studies have examined various stress factors affecting crocodiles, such as stocking densities and environmental stress (8–11).

Corticosterone, a glucocorticoid produced in the adrenal cortex during environmental challenges, is crucial for metabolism, stress response, and adaptation in rodents, birds, reptiles, and amphibians (12). It’s often used as a stress biomarker (13–17). In crocodilian studies, corticosterone levels negatively correlate with growth rates, mortality, immune function, reproductive hormones, and reproductive success (8, 11, 18–21). Plasma corticosterone gauges crocodile stress under various conditions like salinity, water temperatures, capture and restraint methods, and disease (8–10, 22). However, limited research exists on how pen types and stocking densities affect stress. Isberg and Shilton (23) examined group versus individual pens’ effects on saltwater crocodiles’ corticosterone but the experiment did not aim to induce a stress response, but instead was observational in nature.

Stress effects on crocodiles have also been examined using fecal corticosterone levels (24, 25). Fecal samples allow non-invasive glucocorticoid measurements over long periods, providing a better assessment of chronic stress. Furthermore, with the advancement of high-resolution mass spectrometry, we can extend beyond discrete corticosterone measurements and profile the entire cortisol biosynthesis pathway (which includes precursors and intermediate metabolites within the biosynthesis pathway) in order to capture the activated stress response. In previous research on freshwater turtles (*Emydura macquarii macquarii*) we demonstrated that fecal sample metabolome and microbiome assays revealed interactions between the host, gut microbiome, and the environment (26), and that Firmicutes and lower Bacteroidota relative abundances were indicative of stress, as has been observed in other wildlife (27). Here we describe a similar multi-omics approach applied to crocodile faces within a farm context seeking to assess stress and show relationships among glucocorticoids (stress markers), metabolites, and gut microbiota more accurately. To do this, we compared the responses of metabolites and microbiomes between crocodiles in single versus group pens. A targeted and untargeted liquid chromatography-based mass spectrometry (LC–MS) approach was used to measure metabolite, lipid, and hormone profiles in collected faces, while 16S rRNA gene sequencing provided bacterial microbiome community profiles. It was expected that this data would offer a non-invasive method for evaluating stress levels in farmed crocodiles, as it does in other organisms.

## 2 Materials and methods

### 2.1 Animal ethics

This study was conducted under the authority of the CSIRO Wildlife and Captive Large Animals Animal Ethics Committee (CWLA), reference 2020–20, in accordance with the Australian Code for the Care and Use of Animals for Scientific Purposes (28).

### 2.2 Crocodile fecal samples

**Figure 1:**
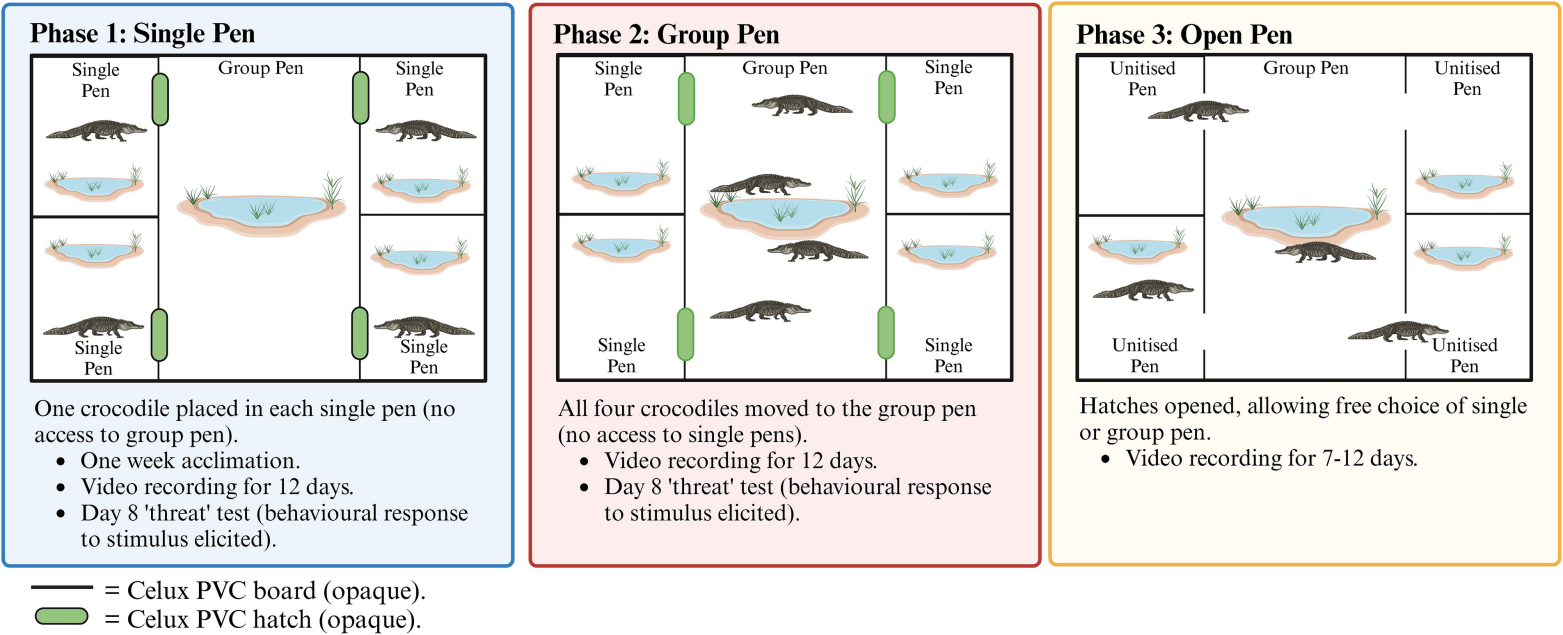
Overview of the study design and three crocodile housing configurations as per Campbell et al. (29).

Samples were collected during the course of the study described by Campbell et al. (29). Briefly, a total of 20 farmed Saltwater crocodiles were housed in groups of 4 under varying conditions, such that each crocodile underwent a period of single housing, followed by housing as the group of 4, and then with free access to both the large group area and the single pen option (Figure 1). Supplementary Figure S1 illustrates the pen configuration. On the morning of day 8, both single- and group-housed crocodiles experienced a physical disturbance (a firm poke in the large muscle of one hind leg with the rounded end of a broom handle) (29), which was performed to trigger a stress response. Following this event, the crocodiles were observed to be ‘*startled*’. All crocodiles used in the experiment were over 3 years old, between 1.5 and 1.9 meters long, and were at the end of their typical finishing period (the final stages of production, when crocodiles are between 1 and 2 m in length).

Fecal samples were collected when available, using a long-handled scoop. Faces were placed into plastic pots (250 mL volume, S10065SL, Labdirect, Wetherill Park, Australia) and frozen at −20°C until analysis. In total, 96 crocodile fecal samples were collected from five assigned groups, over five different sampling times. These groups comprised the following: single (control), single (startled), group (control), group (startled), and preference (post-trial). Table 1 provides a summary of the sample groupings and the associated fecal samples collected per group. Fecal samples were analyzed for metabolites and lipids, corticosterone biosynthesis metabolites, and bacterial 16S rRNA amplicon sequencing, as described below.

**Table 1:** Summary of study design, sample groupings and the number of fecal samples collected per grouping.

Grouping	No. samples	No. time points	Grouping description
Single (Control)	33	5	Single animal pen baseline samples.
Single (Startled)	22	4	Single animal pen after physical disturbance and subsequent days.
Group (Control)	13	4	Multiple animals that have been relocated into a group pen.
Group (Startled)	18	5	Multiple animal pens after physical disturbance and subsequent days.
Preference (post-group trial)	10	3	Phase three, where crocodiles are given free access to the group area and the single pens.

### 2.3 Metabolomics analysis

Metabolites and lipids were extracted from 20 mg freeze dried faces as previously described in Beale et al. (30). Briefly, 20 mg of faces was prepared with 100 μL MilliQ water and 450 μL of ice-cold (−20°C) methanol:ethanol (50% v/v; LiChrosolv®, Merck, Darmstadt, Germany), and vortexed for 2 min. The samples were centrifuged (Centrifuge 5430R, Eppendorf, Hamburg, Germany) at 14,000 rcf at 4°C for 5 min to pellet any protein and solid material. The supernatant was transferred and filtered using a positive pressure manifold (Agilent PPM48 Processor, Agilent Technologies, Santa Clara, California, USA) with Captiva EMR cartridges (40 mg, 1 mL; Agilent Technologies, Mulgrave, VIC, Australia) to separate the lipid and metabolite fraction.

Central carbon metabolism (CCM) metabolites were analyzed on an Agilent 6470 LC-QqQ-MS coupled with an Agilent Infinity II Flex UHPLC system using the Agilent Metabolomics dMRM Database and Method following Sartain (31) and Gyawali et al. (32). Untargeted polar metabolites and non-polar lipids were analyzed using an Agilent 6546 Liquid Chromatography Time-of-Flight Mass Spectrometer (LC-QToF) with an Agilent Jet Stream source coupled to an Agilent Infinity II UHPLC system (Agilent Technologies, Santa Clara, CA, USA) following Shah et al. (33) and Beale et al. (34).

The metabolite and lipid datasets were first filtered and features with >50% missing values per group were removed; remaining missing values were replaced with 1/5 of the minimum positive value of each variable. The data were then log-transformed and multivariate data analysis was conducted using SIMCA (v17.0.01, Sartorius Stedim Biotech, Umeå, Sweden) and MetaboAnalyst 6.0 (35). MetOrigin 2.0 (36) was used to assign to metabolites sources of origin, tied to well-known metabolite databases (i.e., KEGG, HMD, CheBI etc) utilizing the KEGG *Crocodylus porosus* (Australian saltwater crocodile) genome (37) and bacterial 16S rRNA amplicon sequence data to identify host, microbiota, and other metabolite sources. Metabolomics outputs were enriched using Paintomics 4.0 to further explore the contribution of measured biomolecules to corresponding metabolic pathways, which then facilitated a pathway impact assessment (i.e., its criticality in ensuring pathway expression). Significant features were identified using a fold change threshold of ≥2.0 (38) and a Benjamini–Hochberg adjusted *p*-value of ≤0.05 (35).

Two internal standards were used throughout the extraction: 100 ppb of l-Phenylalanine (1-^13^C) and 200 ppb of Succinic Acid (1,4-^13^C_2_). The internal standards were sourced from Cambridge Isotope Laboratories (Andover, MA, USA). The residual relative standard deviation (RSD%) of the internal standards was 8.2% (l-Phenylalanine, 1-^13^C) and 6.5% (Succinic Acid, 1,4-^13^C_2_). Matrix free quality assurance and quality control (QAQC) mixed authentic standards (amino acids and organic acids) and pooled biological quality control (PBQC) samples were analyzed throughout the sequence. QAQC (*n* = 10) and PBQC (*n* = 10) samples were within 5.8–9.6% RSD and 4.2–9.4% RSD, respectively.

### 2.4 Fecal corticosterone analysis

Fecal corticosterone hormones were extracted from 20 mg of faces using Bond Elut Plexa cartridges (30 mg, 1 mL, Agilent Technologies, Mulgrave, VIC, Australia) as per the manufacturer’s instructions. Samples were then separated on an Agilent InfinityLab Poroshell HPH-C8 column (2.1 × 50 mm, 2.7 μm), and analyzed on an Agilent 6546 Liquid Chromatography Time-of-Flight Mass Spectrometer (LC-QToF) with an Agilent Jet Stream source coupled with an Agilent Infinity II Flex UHPLC system. Samples were analyzed in positive electrospray ionization (ESI) using a 1 mM ammonium fluoride mobile phase to improve hormone responses. A 50-ppb heavy-labeled Hydrocortisone-d4 internal standard sourced from Cambridge Isotope Laboratories (Andover, MA, USA) was used (RSD% 2.9). A cortisol biosynthetic pathway Personal Compound Database and Library (PCDL) was created using Masshunter Pathway to PCDL Manager (Version B.08.00, build 8.0.24.0, Agilent Technologies, Santa Clara, USA). The PCDL was sourced from metabolites from known BioCyc/MetaCyc and Wiki pathways (39–41), with MS/MS spectra taken from the Agilent METLIN PCDL (Version 8.0, Agilent Technologies, Santa Clara, USA).

### 2.5 Bacterial 16S rRNA amplicon sequencing

DNA was extracted from 0.25 g freeze-dried fecal material using the DNeasy® PowerSoil® Pro Kit (QIAGEN®; cat. no. 47016) following the manufacturer’s instructions. DNA was eluted in 60 μL of Buffer C6 and quantified on a QuBIT™ Flex Fluorometer with a dsDNA HS kit (Invitrogen™). Negative control extractions were conducted with no starting material and following the same procedure of samples. To investigate changes in the microbiome, we used next generation sequencing of the v1-3 hypervariable region of the bacterial 16S rRNA gene. We used the primers 27F (42) and 519R (43) with Illumina overhang adapter sequences to generate amplicons. PCR reactions consisted of 25 μL GoTaq® Green Master Mix (Promega), 0.2 μM forward primer, 0.2 μM reverse primer, 0.5 μL BSA, and 10–30 ng DNA template in a total volume of 50 μL. Cycling parameters were: denaturation at 95°C x 3 min; 25 cycles of 95°C x 30 s, 55°C x 30 s and 72°C x 30 s; and a final extension at 72°C x 5 min. Amplicon products were purified using Agencourt AMPure XP (Beckman Coulter, Inc., California, USA) as per the manufacturer’s instructions. Purified PCR amplicons were sent to the Ramaciotti Centre for Genomics (UNSW Sydney, Australia) where indexing PCRs to incorporate Nextera XT barcodes, purification, library generation and sequencing were conducted using the Illumina MiSeq platform (with 300 bp paired reads) according to the manufacturer’s directions.

Paired end sequences were merged with flash2 (--min-overlap = 30 --max-overlap = 250) (44). Priming regions were removed from merged reads with cutadapt v2.9 (45). Sequences were then dereplicated and denoised to zero radius operational taxonomic units (zOTU) using USEARCH (46). zOTU abundance table was constructed by mapping all sequences to zOTUs using the USEARCH -otutab command. zOTUs were classified using the silva database (v138) (47–49) and a two-step process. zOTUs were first matched using a consensus method to the silva database (100% similarity cut-off, 100% consensus up to 5 top hits). Any sequences not classified with this method were classified using the QIIME2 (50) sk-learn Bayesian classifier (--p-confidence 0.6). Putative contaminants were removed from the abundance table using the Decontam R package (51) with the “prevalence” method and threshold = 0.5, as suggested by the documentation to “identify as contaminants all sequences that are are more prevalent in negative controls than in positive samples.” Finally, any sequences not classified as “bacteria,” unclassified at the Phylum level, classified as “Chloroplast” or “Mitochondria” were removed. The data were filtered with a minimum median OTU abundance threshold of 4 reads, and a variance threshold of 10% based on the inter-quartile range. Rarefaction curves reached saturation at 2732 sequences per sample indicating a sufficient sampling depth was achieved. Data was then transformed using the centered log ratio (CLR) method. A dysbiosis score based on median community level variation was performed after Lloyd-Price et al. (52).

## 3 Results and discussion

### 3.1 Metabolic profile of naïve farmed crocodiles (control samples)

Overall, 94 CCM metabolites, 238 polar metabolites and 232 non-polar lipids were identified across all crocodile fecal samples resulting in 564 metabolic features. Of the identified features, 15 metabolites were annotated as belonging to the cortisol biosynthesis pathway. An overview of the metabolite and lipid chemical class characterization within the crocodile fecal samples is presented in Supplementary Figure S2. The fecal samples show a high presence of ceramide non-hydroxyfatty acid-sphingosine (25.23%) and other lipids like hexosylceramide, sulfatide, and free fatty acids. Among non-lipid metabolites, amino acids and peptides are the most dominant (14.15%), followed by fatty acids and conjugates (11.38%), and monosaccharides (6.46%). Other groups make up less than 5% each (see Supplementary Table S1).

**Figure 2:**
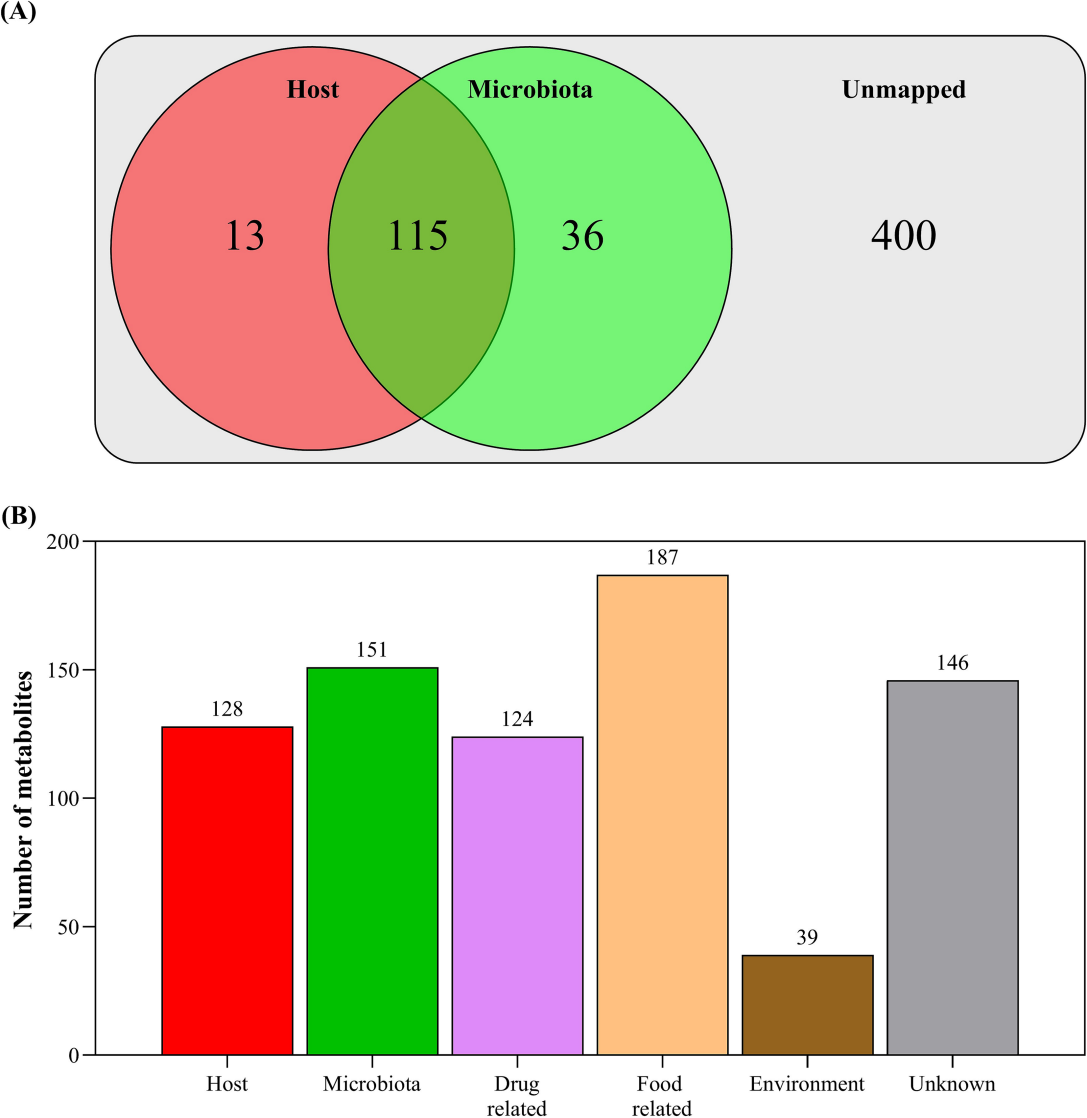
**(A)** Annotated metabolites that were mapped to host or microbiota origins based on known metabolic pathways within the KEGG *Crocodylus porosus* (Australian saltwater crocodile) genome (37) and 16S bacterial rRNA amplicon sequence data; and **(B)** annotated metabolites that were mapped to origin sources based on publicly available metabolite databases within MetOrigin 2.0 (http://metorigin.met-bioinformatics.cn/).

Based on the metabolite origin analysis (Figure 2A), 128 metabolites were identified as originating from the host crocodiles (13 unique to the host), 151 metabolites were identified as originating from their associated microbiota (36 unique to the microbiota) and the origin of the remining 400 metabolites could not be assigned. The matched metabolites were then classified as belonging to a range of biological processes/sources, with some overlap between categories (Figure 2B). The host-related metabolites were principally annotated to metabolism (79.27%), organismal systems (4.88%), cellular processes (7.32%), environmental information processing (6.10%), and genetic information processing (2.44%).

Across the analyzed biomolecules, with respect to the control, single and initial group pen fecal samples, no metabolites or lipids were significantly altered. This indicates that the baseline control samples were statistically similar to the single pen samples and the initial group pen samples ([PERMANOVA] *F*-value: 0.067852; R-squared: 0.0012549; *p*-value (based on 999 permutations): 0.946). This is graphically presented in Supplementary Figure S3. In agreement to this, Isberg and Shilton (23) did not observe a significant difference in stress level between crocodiles from group pens or individual pens by comparing the plasma corticosterone levels of the two groups. The similar metabolic profile of control crocodiles in two pen types allows for a further comparison of startle stress responses of animals in single pens versus those in group pens.

### 3.2 Fecal corticosterone (stress hormone) and the cortisol biosynthetic pathway

**Figure 3:**
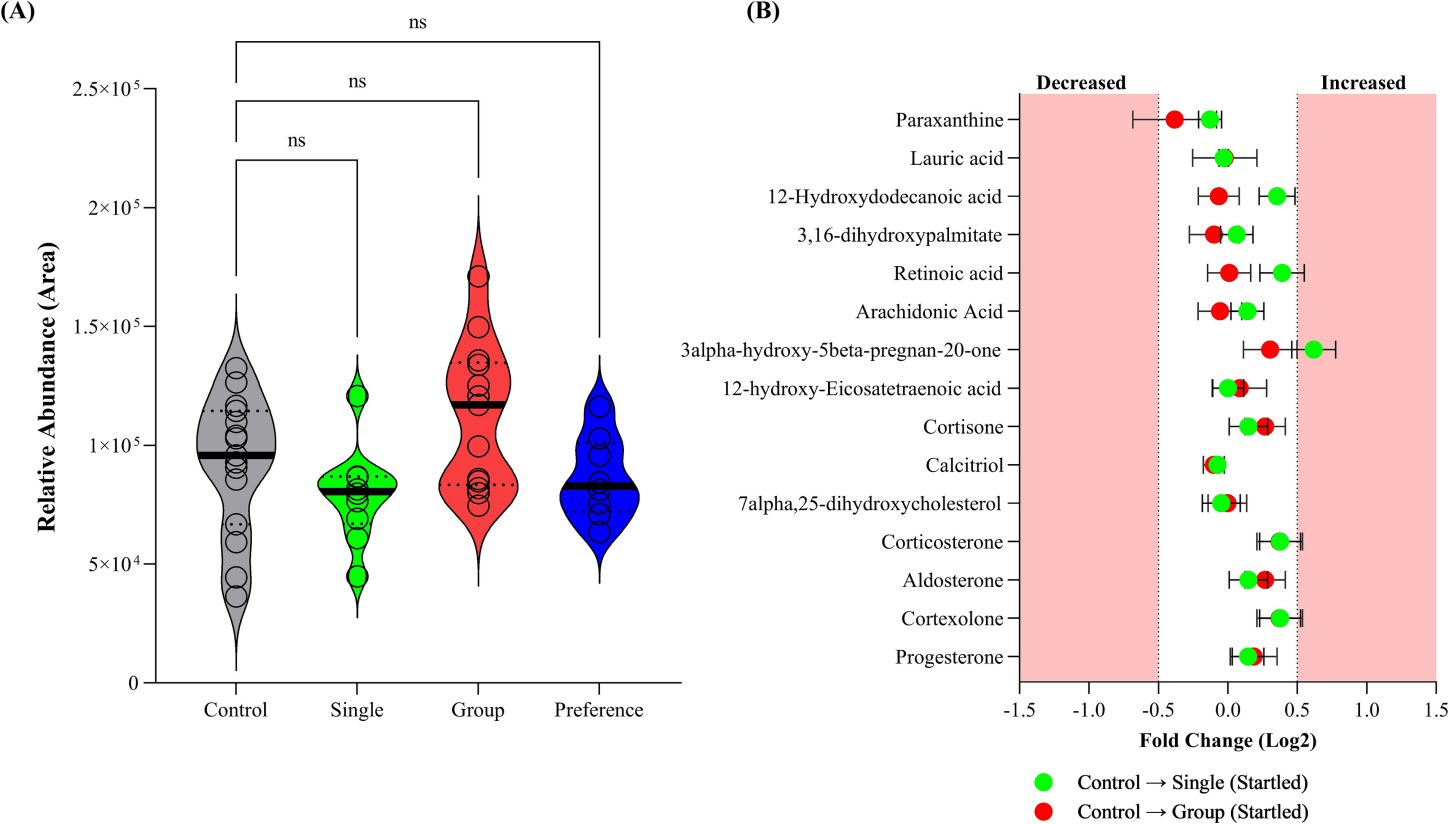
**(A)** Corticosterone levels within analyzed crocodile feaces collected from control, single (startled) and group (startled) pens; and **(B)** fold Change of cortisol biosynthesis pathway metabolites in fecal samples collected from startled single and startled group crocodiles with respect to the control group.

Fecal corticosterone levels measured in the collected faces from single and group penned crocodiles showed no significant differences between the sampled groups (Figure 3A), aligning with the plasma corticosterone results documented by Isberg and Shilton (23). While not statistically significant, the mean relative abundance of the Dunnett’s multiple comparisons test was lower for the single and preference group pens at 79,312 (adjusted *p*-value = 0.3308) and 86,273, respectively (adjusted *p*-value = 0.8038), when compared to the control group (94,748). Conversely, the group pen crocodiles were observed to have an elevated mean relative corticosterone abundance (112,283; adjusted *p*-value = 0.1784). However, interpretation of single-time-point corticosterone levels *per se*, as an indicator of stress, is fundamentally flawed. Corticosterone and cortisol have been observed to be elevated in other non-stressed related circumstances, such as positive arousal situations (53–55).

The cortisol biosynthetic pathway has been identified as the most affected pathway in plasma following hypoxia and re-oxygenation (56, 57). In the present study, the cortisol biosynthesis pathway metabolites in faces within three crocodile sample groups were not statistically different based on fold change (Figure 3B). This is indicative of inactivated cortisol biosynthetic pathway and the intermediate stress hormone, corticosterone, not being produced, suggesting no stress was observed in these animals.

It is proposed that fecal corticosterone reflects more stable and long-term stress levels, while blood corticosterone varies more, tends to spike and signifies a short-term stress response (58). Importantly, the host microbiome, environmental conditions, and male and female hormonal status are known to alter fecal corticosterone levels and impact their interpretation (58). The gut microbiome is essential in controlling the host’s endocrine system and stress response, influenced by sex hormones like estrogen and testosterone, which are further affected by external stressors (59–61). To overcome these limitations and provide greater insight to the crocodile stress response, metabolomics and microbial community profiling of fecal samples was performed.

### 3.3 Metabolic variations between single and group pens during the startle test

**Figure 4:**
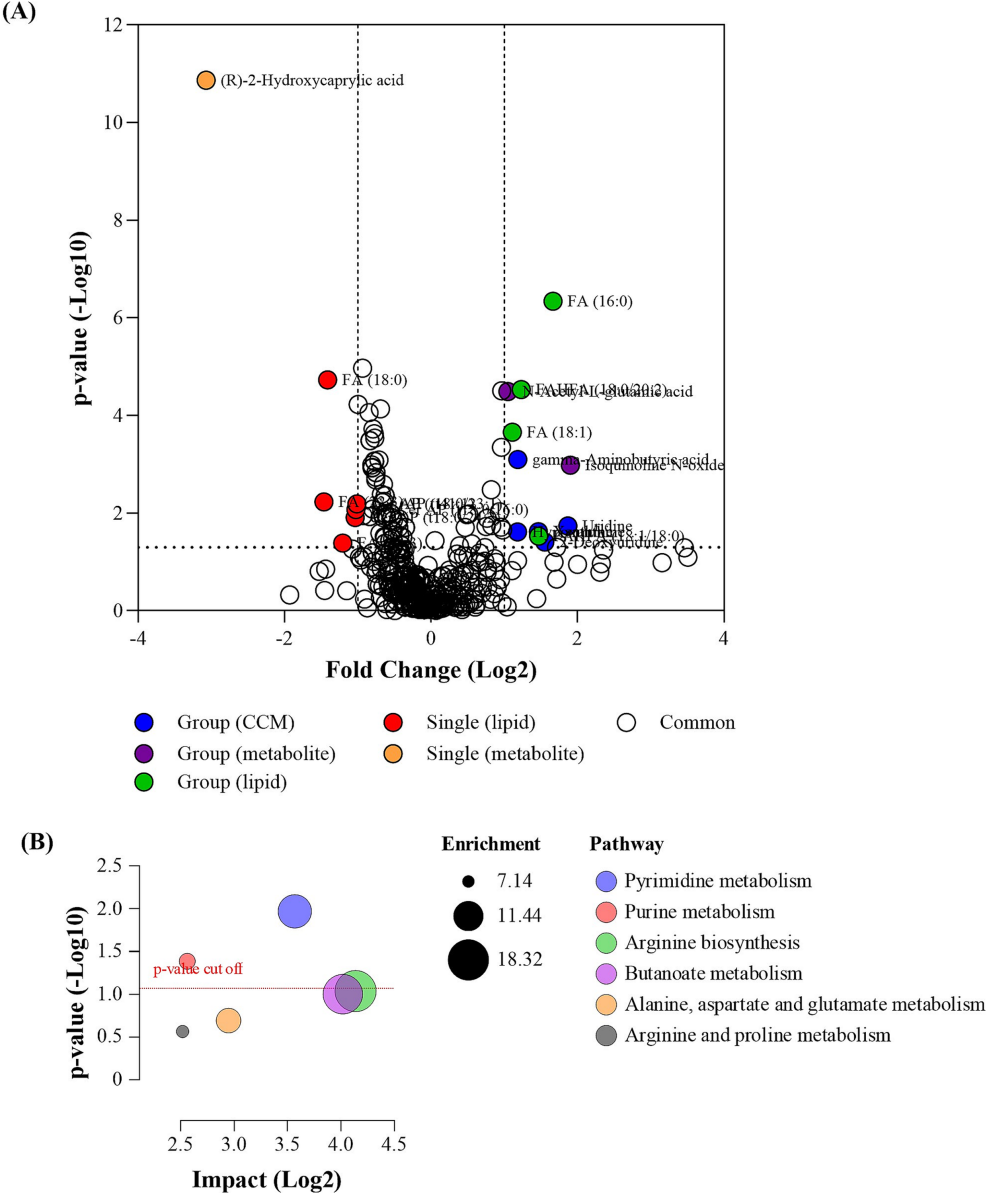
**(A)** Volcano plot of the analyzed biomolecules in the sampled faces collected from single animal pen and group animal pen post stressor; and **(B)** pathway enrichment and impact plot of significant metabolites identified between the single startled and group startled crocodile groups.

A pairwise comparison between single and group pen arrangements was conducted to assess the metabolic responses of animals in each pen type that was subjected to a stress test. Multi-variate analysis was employed to identify biomolecules responsible for differences between the groups, with PLS-DA score plots (Supplementary Figure S4) highlighting distinct distribution variances in measured biomolecules, particularly polar metabolites, indicating differing metabolic responses between the two groups. Notably, 21 features were statistically different (Figure 4A; Supplementary Table S2), and among these, single pen crocodiles exhibited a downregulation of 9 compounds and an upregulation of 12 compounds when compared to group crocodiles. These metabolic differences resulted in enriched pyrimidine metabolism and purine metabolism pathways being impacted (Figure 4B), which are linked to altering energy pathways that have been associated with various animal models exposed to chemical stressors (62).

**Figure 5:**
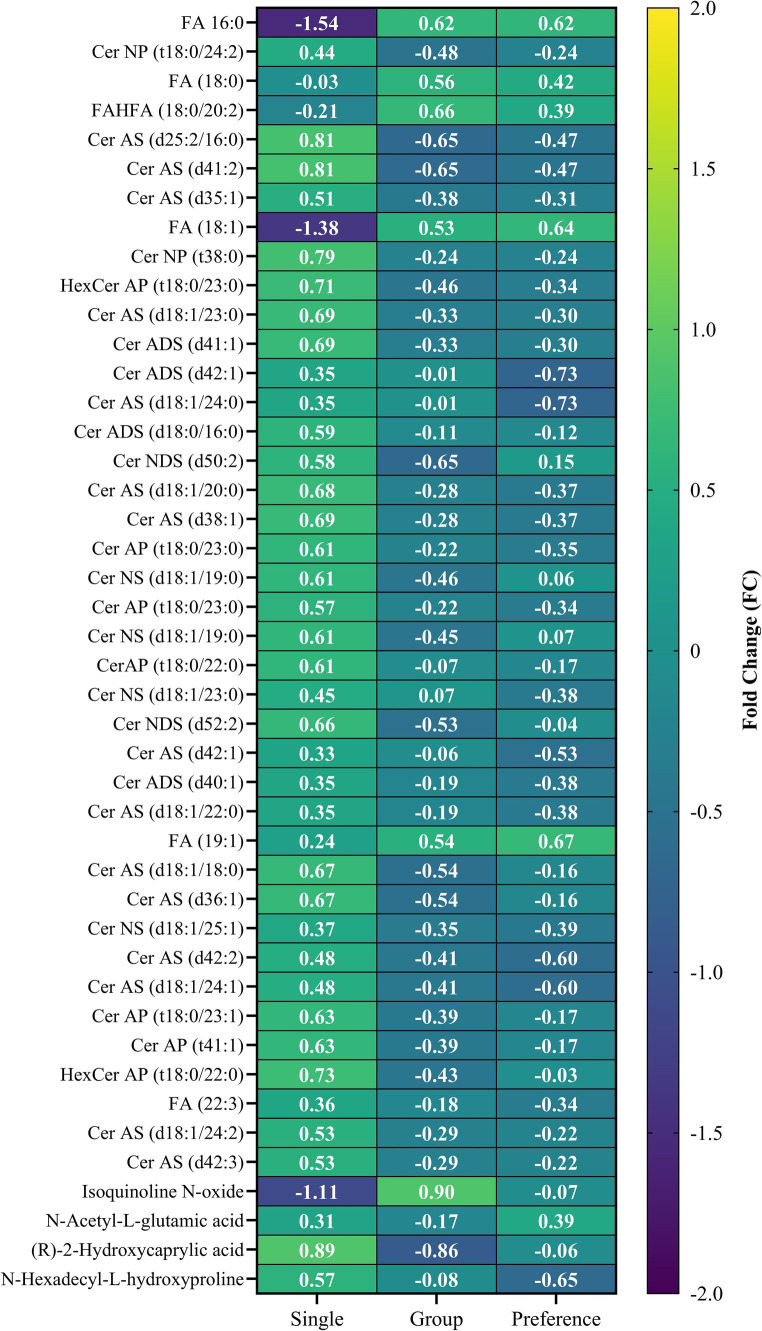
Heatmap of biomolecules that were significantly different between the single and group animal pen samples and the preference animal samples collected at the end of the trial.

The startle test significantly impacted fecal 2-Hydroxycaprylic acid, which was found at lower levels in the single pen crocodiles compared to those in the group pens (Figure 5). Known as D-2-Hydroxyoctanoic acid, this medium-chain fatty acid has been studied for its various properties (63). In bovine research, a diet supplemented with *Perilla frutescens* leaf (PFL) led to decreased 2-hydroxycaprylic acid in cow’s milk relative to a control diet (64). This reduction showed a negative correlation with ruminal deoxycytidine and a positive correlation with ruminal uridine 5-monophosphate. PFL is recognized for its antibacterial, anti-inflammatory, and antioxidant properties due to its bioactive compounds (65–67). These findings suggest that the reduced levels of 2-hydroxycaprylic acid might be linked to cow health and metabolism. Additionally, 2-hydroxycaprylic acid was identified as one of twelve down-regulated differential metabolites associated with the survival of patients with Gastric Cardia Adenocarcinoma (GCA), a malignant tumor (68). Therefore, the lowered levels of 2-Hydroxycaprylic acid in single pen crocodiles during the startle test could indicate an altered, possibly improved, health condition compared to those in group pens.

Another down-regulated metabolite in single pen crocodiles was dopamine, a crucial neurohormone of the sympathoadrenal system (69). Since dopamine influences behavior (70), its differing levels between the two pen types suggest varied responses to rotational stress. Increased dopamine can signal induced stress (71), and its fluctuation alongside other energy metabolites (lipids) in single pen crocodiles supports this notion. Dopamine also plays a vital role in thermoregulation (72–74) explaining its higher levels in group pen crocodiles, who spent less time in water regulating their body temperature (29). Collectively, the lower levels of 2-Hydroxycaprylic acid and dopamine in single pen crocodiles implies these animals experienced less physiological arousal compared to group pen crocodiles (29). The daily activity patterns were more uniform in group pens than in single pens (29), which was attributed to the presence of a dominance hierarchy, under which the more subordinate animals may not have been able to perform preferred behaviors or access preferred locations. This could have resulted in anxiety.

Single pen crocodiles had higher levels of compounds like *γ*-Aminobutyric acid, uridine, xanthine, and hypoxanthine compared to those in group pens, potentially due to different stress responses. γ-Aminobutyric acid (GABA) is a well-known neurotransmitter that reduces stress and enhances sleep (75). GABA accumulation in response to environmental stress has been seen in both plants (76, 77) and animals (78, 79). Uridine, a pyrimidine nucleoside, functions in the central nervous system (80, 81). Its increase in plasma during physical exercise, ethanol ingestion, fructose infusion, and xylitol infusion enhances adenine nucleotide degradation, raising plasma purine base concentrations (82–84). The observed rise in xanthine and hypoxanthine alongside uridine could result from the stress tests, indicating better stress regulation in single pen crocodiles.

In fecal samples of single pen crocodiles, we observed an increase in *N*-acetylglutamic acid, which is produced from glutamic acid and acetyl-CoA by the *N*-acetylglutamate synthase (NAGS) enzyme, and is an essential activator of carbamoyl phosphate synthetase (CPSI) in the urea cycle within the mitochondrial matrix. Its accumulation has been noted in bipolar disorder patients, suggesting mitochondrial dysfunction (85). However, its decrease were observed in *Bombyx mori* after NaF stress (86). The role of increased *N*-acetylglutamic acid in fecal samples of single pen crocodiles under stress remains unclear and needs further research.

### 3.4 Metabolic changes post animal stress

**Figure 6:**
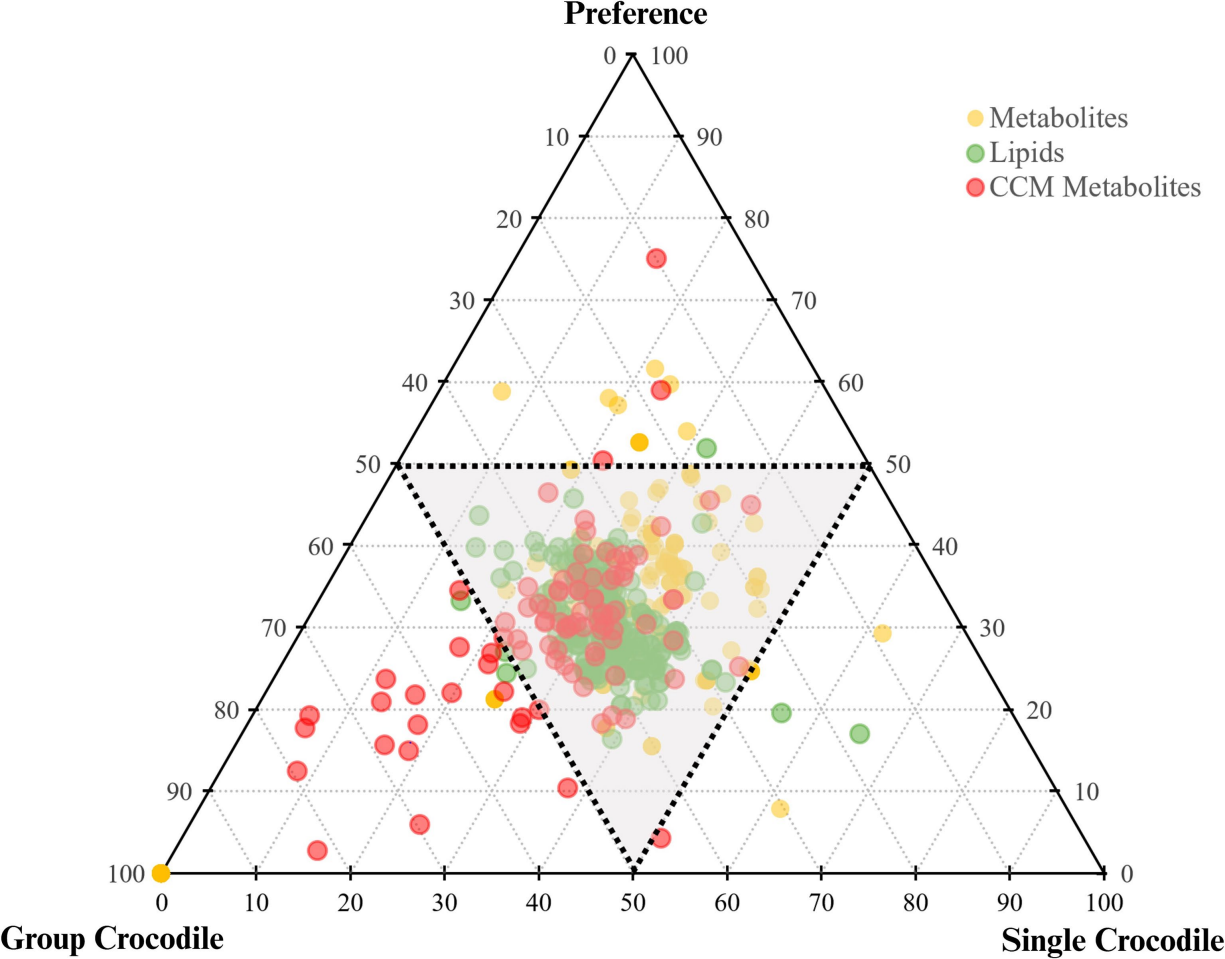
Ternary plot of the analyzed crocodile fecal samples from the single (startled), group (startled) and the post-trial preference for isolation groups.

At the end of the stress trial, crocodiles in group pens could choose to isolate. In the behavioral study, some animals chose to isolate, but it was unclear whether the motivator to utilize the single pens was isolation or access to an under-shelf area (29). To assess whether the metabolic profile of group pen crocodiles reverted to that of single-pen crocodiles after provision of the opportunity to isolate, a three-way comparison was performed. Figure 6 shows a ternary plot highlighting key biomolecules driving differences among these groups. Supplementary Table S3 lists features identified by one-way ANOVA (Supplementary Figure S5). There were 43 significantly different features (4 polar metabolites and 39 lipids), primarily between single pen and group pen crocodiles. The heatmap (Figure 5) showed most lipids were higher in single pen crocodiles, suggesting more energy reserves. The group animals differed from the single preference animals in only four compounds (Ceramide ADS d42:1, Ceramide AS d18:1/24:0, N-Acetyl-L-glutamic acid, N-Hexadecyl-L-hydroxyproline). Of these, N-Acetyl-L-glutamic acid, N-Hexadecyl-L-hydroxyproline increased when crocodiles moved out of the group pen and isolated (preference group) while two lipids (Ceramide ADS d42:1, Ceramide AS d18:1/24:0) decreased. This may indicate some slight change in physiology and metabolism of crocodiles from group pens after they were released from the group pens, but the biological background remains unknown and needs future investigation. Additionally, lipid increases in preference group crocodiles could be due to recovery from startle testing.

### 3.5 Microbiome and the Firmicutes:Bacteroides (F:B) as an indicator of stress

**Figure 7:**
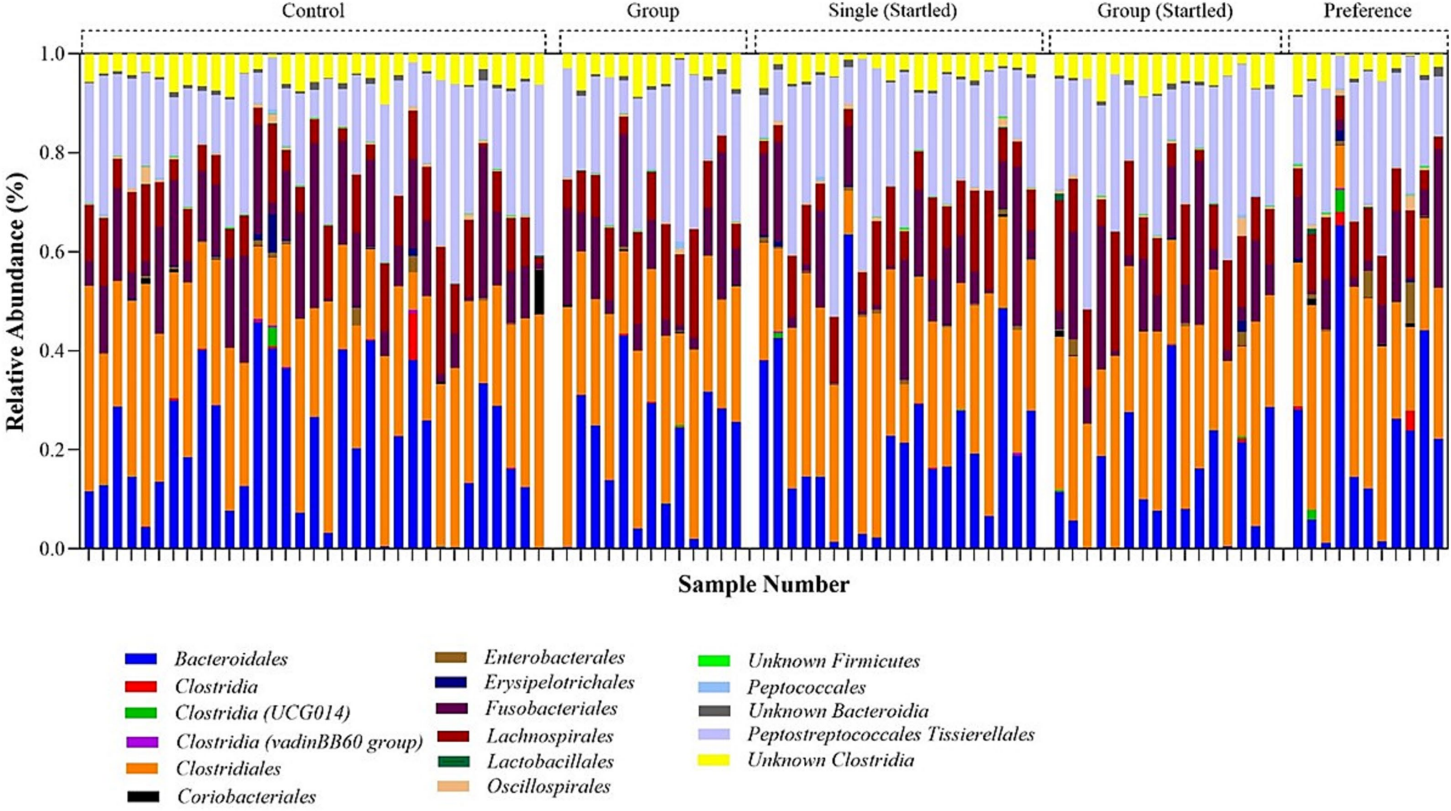
Relative bacterial order abundance in the sampled crocodile faces obtained from 16S rRNA bacterial amplicon sequencing.

The quantity of quality-filtered sequences obtained for each sample ranged from 2,735 to 65,860, culminating in a total of 3,507,013 sequences (with an average of 36,915 reads per sample). Data filtering applied a minimum median operational taxonomic units (OTU) abundance threshold of 4 reads and a variance threshold of 10% based on the interquartile range. All rarefaction curves achieved saturation (Supplementary Figure S6), indicating that sufficient sampling depth was reached to adequately represent the community diversity in each sample at a rarefied library size of 2,735 sequences. Figure 7 presents an overview of the microbial community’s relative abundance at the Order level. Supplementary Figures S7A,B display the alpha diversity (Chao1) for the single (control), group (control), single (startled), group (startled), and preference groups. The *p*-value t-tests for Chao1 were not significant (*p*-value >0.05), indicating a normally distributed and homogenous microbial community. This is anticipated for organisms in a farmed environment where the diet consists of a uniform food supply. The beta diversity analysis, shown as a Principal Coordinates Analysis (PCoA) plot based on Bray-Curtis distances in Supplementary Figure S7C, does not exhibit separation on the ordination plot, signifying consistent bacterial communities across the sample groups.

**Figure 8:**
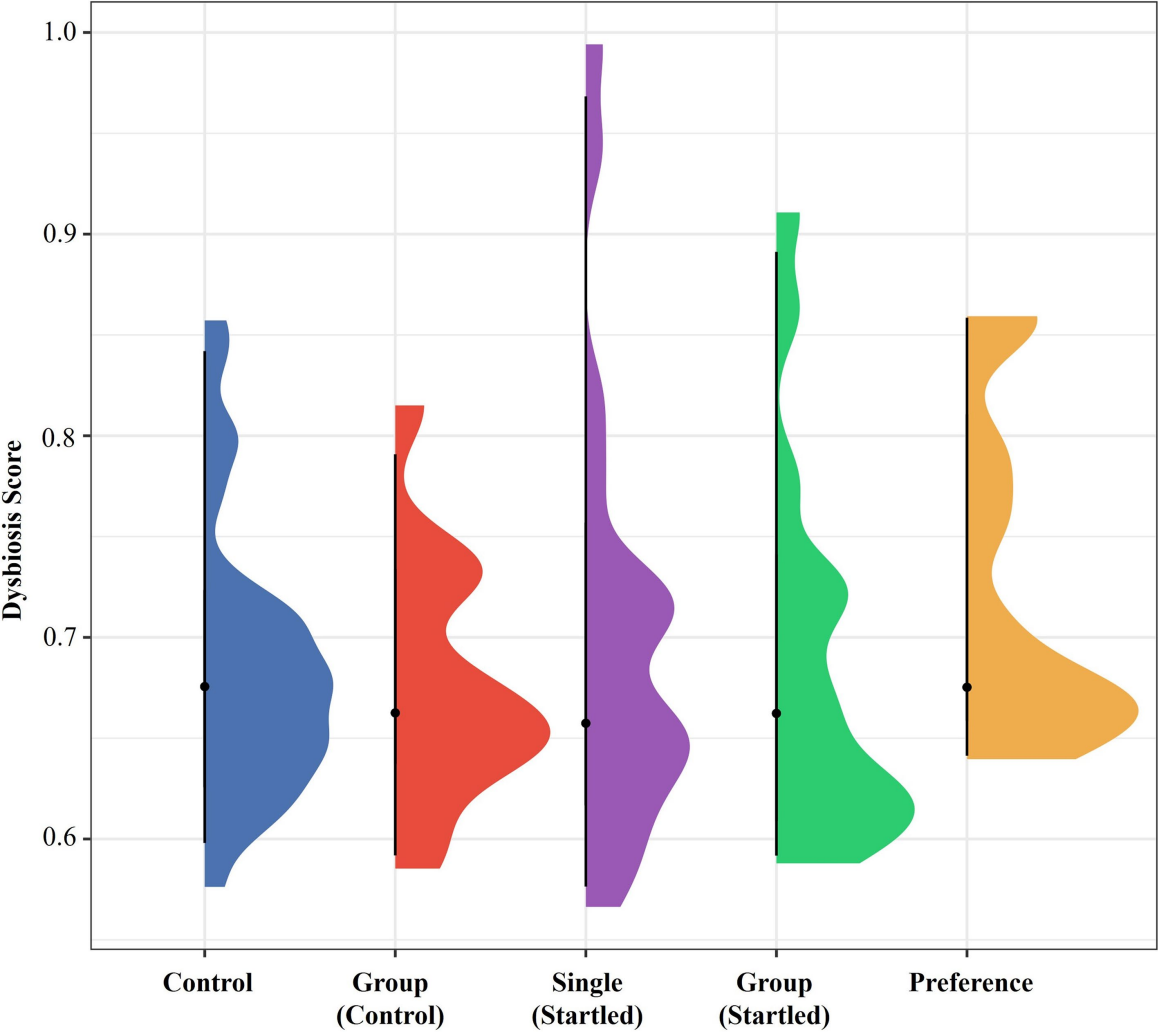
Violin plot showing the distribution of Dysbiosis Scores across five sample classes: Control, Group (control), Single (startled), Group (startled), and Preference. The Dysbiosis Score reflects microbial community imbalance, with higher scores indicating greater dysbiosis. The width of each violin represents the density of samples at each Dysbiosis Score, while the black dot and line indicate the median and interquartile range for each class. The black dots within each plot indicate the median Dysbiosis Score, while the vertical black lines represent the interquartile range (IQR), capturing the middle 50% of the data.

Figure 8 further investigates the microbial community dynamics by displaying the dysbiosis scores across five different sample classes: control, group (control), single (startled), group (startled), and preference. The presence of similar dysbiosis scores across the different sample classes suggests a relatively consistent level of microbial imbalance among them. The observed homogeneity in dysbiosis scores aligns with the alpha and beta diversity results, reinforcing the conclusion that microbial communities within these sample classes are stable and uniform, potentially due to the controlled diet and environment typical of farmed organisms.

**Figure 9:**
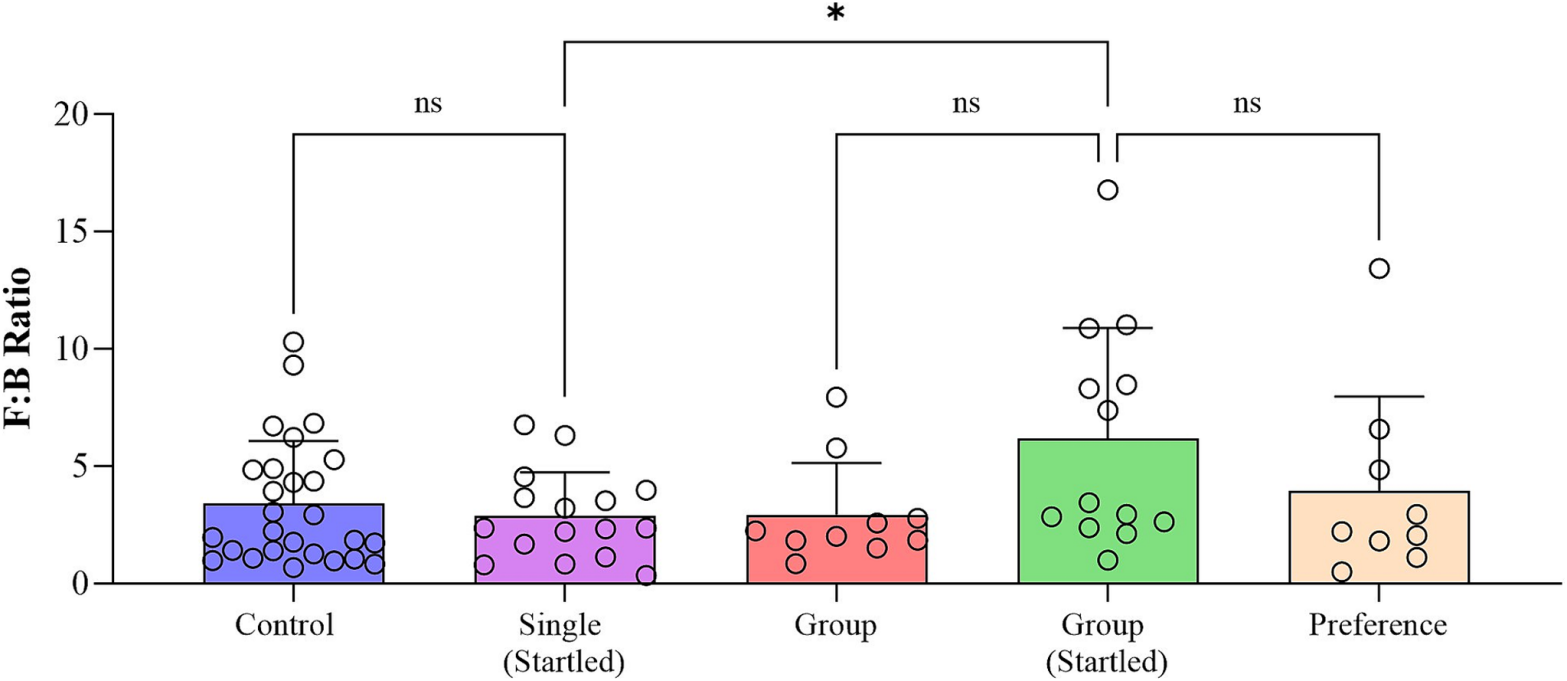
Fecal microbiome Firmicutes:Bacteroides (F:B) ratio in each phase of the study (Boxplot represents all datapoints in each phase of the study with statistical outliers removed following the ROUT outlier method based on the False Discovery Rate (FDR) of Q% = 1).

The analysis of the bacterial community profile identified five main components of the microbiota in crocodile fecal samples, including Firmicutes, Bacteroidota, Proteobacteria, Fusobacteria, and Actinobacteria. Among these, Firmicutes (65.1–74.0%) were the most abundant group followed by Bacteroidota (14.8–23.0%), and Fusobacteriota (8.1–12.9%), which together comprised 98.2–99.8% of the total relative microbiome sampled. Fecal microbiome results also indicated that animals in the single pens were more able to cope with a stressful event than animals in the group pen, as evident in the significant increase in Firmicutes:Bacteroides (F:B) ratio (an indicator of stress) following the startle test in group housed animals, but not in the single housed animals (Figure 9). There is also a suggestion that group housed animals might slowly become more stressed/anxious as time passes, and this is maintained into the free-choice phase (possibly due to their continued group confinement despite having more opportunities to distance themselves from others).

**Figure 10:**
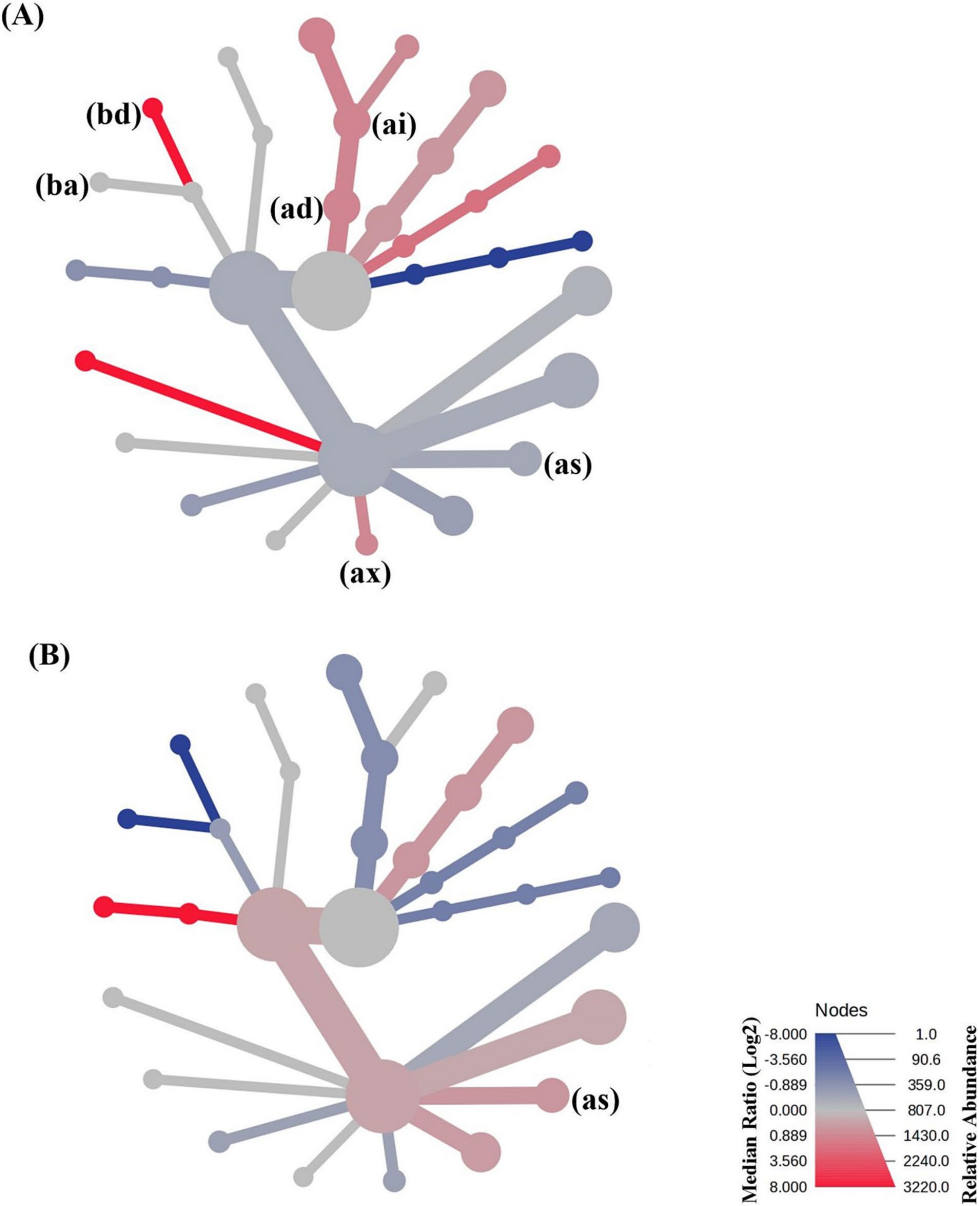
Heat tree analysis plot of taxonomic differences between the microbial community members within **(A)** the single startled cohort and group startled cohort of crocodiles, and **(B)** the group startled cohort, and the preference cohort of crocodiles sampled. Note that (bd) is Erysipelotrichales; (ba) is Lactobacillales; (ai) is Bacteroidota; (ad) is Bacteroidia; (as) is Unknown Clostridia; and (ax) is Oscillospirales.

We conducted a heat tree analysis that leverages the hierarchical structure of taxonomic classifications to quantitatively (using the median abundance) and statistically (using the non-parametric Wilcoxon Rank Sum test) depict taxonomic differences between microbial communities (87). The result from this statistical comparison is presented in Figure 10.

Differences between the single startled cohort and the group startled cohort include a relative increase in Erysipelotrichales (bd; *p* = 0.0295), Bacteroidota (ai; *p* = 0.0950), and Bacteroidia (ad; *p* = 0.0949); and a decrease in Unknown Clostridia (as; *p* = 0.0855) and Oscillospirales (ax; *p* = 0.0426) in the group startled cohort of crocodiles compared to the single startled cohort. However, when the group cohort were given a preference to isolate or not, there was an increased abundance in the Unknown Clostridia members (as; *p* = 0.07155).

Increases in Erysipelotrichales have been linked to the onset of cancer in humans, and decreases have been associated with Crohn’s and IBS (88). However, perturbation of Erysipelotrichales has been reported to be associated with host metabolic disorders and inflammatory diseases (89), which could explain an increase in the group startled cohort herein and its association with elevated stress. Oscillospirales were significantly correlated with short-chain fatty acids and lipid metabolism (90), which was perturbed in the group startled cohort. The interplay between Clostridia, the microbiome, and stress in wildlife has been studied (91, 92). Clostridia is an order of bacteria in the gut microbiome that significantly influences various physiological processes like stress responses (93), and was observed to be increased in the preference cohort crocodiles when compared to the group startled cohort.

Stress greatly affects the gut microbiome’s makeup and function. Gut bacteria like Clostridia can impact the body’s stress responses by influencing neurotransmitters such as serotonin and GABA, key for mood regulation. Clostridia also affect the hypothalamic–pituitary–adrenal (HPA) axis, the central system for stress response (94). The fecal microbiome of the group (startled) crocodile cohort generally suggested a potential stress response, evident by the increased presence of Clostridia and other taxa. These findings align with other data that shows the group cohort experienced higher stress levels when startled compared to single crocodiles. Prolonged exposure to heightened stress conditions could lead to gut dysbiosis (95). Further research is necessary to assess this possibility within a farm setting and its implications for crocodile production.

The analysis of metabolic pathways in various animal pen samples throughout the trial revealed significant enrichment in several pathways (Supplementary Table S4). Notably, purine metabolism showed significant differences between the control and single startled groups (*p* = 0.045) and between group startled and preference groups (*p*-value = 0.023). Propanoate metabolism was significantly different between control and single startled groups (*p*-value = 0.019) and between single startled and group startled groups (*p*-value = 0.048). Glycolysis/gluconeogenesis and glycerolipid metabolism had significant differences in multiple comparisons. These pathways are important for regulating glucose levels during stress. Stress enhances gluconeogenesis and glycolysis to fulfill energy requirements, which may lead to metabolic problems if sustained over a long period (96). Additionally, methane metabolism, phenylalanine, tyrosine, and tryptophan biosynthesis, and thiamine metabolism were significantly enriched in the single startled vs. group startled comparison. The production of methane within the body is associated with oxidative stress responses (97). Stress can influence the biosynthesis of phenylalanine, tyrosine, and tryptophan, as these amino acids are precursors to neurotransmitters such as serotonin and dopamine that are essential for mood regulation (98). Additionally, stress can lead to a depletion of thiamine levels, resulting in neurological and psychiatric symptoms (99). These findings highlight the impact of different environmental conditions on metabolic pathways in animal samples.

An analysis of the correlation between metabolites and microbial members (Order) in animal pen samples during the trial identified several significant relationships (Supplementary Table S5). For instance, Enterobacterales showed a correlation with dihydroxyacetone phosphate (R^2^ = 0.294, *p* = 0.056) and hypoxanthine (R^2^ = 0.283, *p* = 0.066) in the Control vs. Single Startled group. Dihydroxyacetone phosphate is involved in glycolysis and gluconeogenesis, which are critical for energy production (100). Hypoxanthine is a metabolite involved in purine metabolism and can act as a stress marker (101). Its correlation with Enterobacterales suggests that these bacteria might influence stress responses by modulating energy metabolism and cellular stress pathways. Additionally, Unknown Clostridia was correlated with N-acetylneuraminic acid (R^2^ = −0.022, *p* = 0.887) in the same group. In the Group Startled vs. Preference comparison, galactonic acid was negatively correlated with microbial orders (R^2^ = −0.324, *p* = 0.114), while hypoxanthine showed a positive correlation (R^2^ = 0.165, *p* = 0.431). For the Single Startled vs. Group Startled group, 4-guanidobutyric acid and D-gluconic acid showed correlations with microbial orders, with R^2^ values of −0.211 (*p* = 0.239) and 0.204 (*p* = 0.256), respectively. D-Gluconic acid is involved in carbohydrate metabolism and 4-Guanidobutyric acid is involved in amino acid metabolism, impact stress responses by affecting neurotransmitter levels and energy metabolism (102, 103). These correlations suggest possible associations between these bacteria and metabolites, although none are statistically significant. This implies that while there may be some patterns, they are not strong enough to draw definitive conclusions about their relationships under stressful conditions.

## 4 Conclusion

The study investigates the metabolic stress in single and group housed farmed saltwater crocodiles (*C. porosus*) by analyzing their fecal metabolome and microbiome. Under the parameters of the current study, group housing appears to induce an increased stress response in the studied crocodiles compared to the single pen system, specifically:

- Crocodile faces comprise a complex mixture of metabolites associated with a range of metabolic activities. The study identified 564 metabolic features within the analyzed fecal samples. Among these, 15 metabolites were annotated as part of the cortisol biosynthesis pathway.
- Crocodile fecal metabolites originate from different sources. Our analysis revealed that 128 metabolites originated from the host crocodile, 151 from the microbiota, and 400 metabolites could not be matched to their origin. The host-related metabolites were primarily associated metabolism (79.27%), organismal systems (4.88%), cellular processes (7.32%), environmental information processing (6.10%), and genetic information processing (2.44%).
- Conventional stress response measures found no significant differences in fecal corticosterone levels between single and group penned crocodiles, indicating no stress was observed in these animals. However, the mean relative abundance of corticosterone was lower for single and preference group pens compared to the control group.
- Conversely (to corticosterone levels), metabolic variations between single and group housed crocodiles were observed and associated with stress. A pairwise comparison between single and group pen arrangements showed distinct metabolic responses to stress. Single pen crocodiles exhibited a downregulation of 9 compounds and upregulation of 12 compounds compared to group crocodiles. These differences impacted pyrimidine metabolism and purine metabolism pathways, which are linked to altering energy pathways.
- Changes to the microbiome community between individual and group housing arrangements revealed a higher Firmicutes:Bacteroides (F:B) ratio in the fecal microbiome of group-housed saltwater crocodiles. This elevation is indicative of increased stress and is corroborated by a greater relative abundance of Clostridia taxa, which commonly rises in the gut under stress conditions.

These findings suggest that the fecal metabolome and microbiome can provide additional insights into the metabolic stress and overall health of farmed saltwater crocodiles. Furthermore, these findings support those of Campbell et al. (29), which indicated that the presence of a dominance hierarchy in the group pen may have had negative impacts on the animals. Further work is required to understand if this approach can be applied to different stressors, different species and different age groups of crocodilians, and to develop the approach to a point at which it can be utilized to guide management decisions.

## Data Availability

The original contributions presented in the study are included in the article/Supplementary material, further inquiries can be directed to the corresponding author.

## References

[1] Lott MJ Moore RL Milic NL Power M Shilton CM Isberg SR. Dermatological conditions of farmed crocodilians: a review of pathogenic agents and their proposed impact on skin quality. Vet Microbiol. (2018) 225:89–100. doi: 10.1016/j.vetmic.2018.09.022, PMID: 30322539

[2] Isberg S Thomson P Nicholas F Webb G Manolis S Barker S. Quantitative analysis of production traits in saltwater crocodiles (*Crocodylus porosus*): IV. Number of scale rows. J Anim Breed Genet. (2006) 123:48–55. doi: 10.1111/j.1439-0388.2006.00561.x PMID: 16420265

[3] MacNamara K Nicholas P Murphy D Riedel E Goulding B Horsburgh C. Markets for skins and leather from the goat, emu, ostrich, crocodile and camel industries. Publica. (2003) 2:142. Available at: https://webarchive.nla.gov.au/awa/20030512063546 (Accessed January 22, 2025).

[4] Thorbjarnarson J. Crocodile tears and skins: international trade, economic constraints, and limits to the sustainable use of crocodilians. Conserv Biol. (1999) 13:465–70. doi: 10.1046/j.1523-1739.1999.00011.x

[5] Saalfeld K Fukuda Y Duldig T Fisher A. Management program for the saltwater crocodile (*Crocodylus porosus*) in the Northern Territory of Australia, 2016–2020. Darwin: Northern Territory Department of Environment and Natural Resources (2016).

[6] Webb GJW Reynolds S Brien ML Manolis SC Brien JJ Christian K. Improving Australia’s crocodile industry productivity. Agrifutures Australia. (2013). Available at: https://www.agrifutures.com.au/product/Improving-Australia-s-Crocodile-Industry-Productivity/ (Accessed January 22, 2025).

[7] Isberg S Thomson P Nicholas F Barker S Moran C. (2004). Farmed saltwater crocodiles: a genetic improvement program. Rural Industries Research and Development Corporation Paper.

[8] Elsey RM Joanen T McNease L Lance V. Stress and plasma corticosterone levels in the American alligator—relationships with stocking density and nesting success. Comp Biochem Physiol A Physiol. (1990) 95:55–63. doi: 10.1016/0300-9629(90)90009-H

[9] Laurén DJ. The effect of chronic saline exposure on the electrolyte balance, nitrogen metabolism, and corticosterone titer in the American alligator, *Alligator mississippiensis*. Comp Biochem Physiol A Physiol. (1985) 81:217–23. doi: 10.1016/0300-9629(85)90125-2, PMID: 2864163

[10] Nevarez JG Lattin CR Romero M Stacy B Kinler N. Assessment of corticosterone levels in American alligators (*Alligator mississippiensis*) with dermatitis. J Herpetol Med Surg. (2011) 21:76–9. doi: 10.5818/1529-9651-21.2.76

[11] Turton J Ladds P Manolis S Webb G. Relationship of blood corticosterone, immunoglobulin and haematological values in young crocodiles (*Crocodylus porosus*) to water temperature, clutch of origin and body weight. Aust Vet J. (1997) 75:114–9. doi: 10.1111/j.1751-0813.1997.tb14170.x, PMID: 9066968

[12] Koob GF. Encyclopedia of behavioral neuroscience. Amsterdam: Elsevier (2021).

[13] Badmus KA Idrus Z Meng GY Sazili AQ Mamat-Hamidi K. Telomere length and regulatory genes as novel stress biomarkers and their diversities in broiler chickens (*Gallus gallus* domesticus) subjected to corticosterone feeding. Animals. (2021) 11:2759. doi: 10.3390/ani11102759, PMID: 34679783 PMC8532957

[14] Cook NJ. Minimally invasive sampling media and the measurement of corticosteroids as biomarkers of stress in animals. Can J Anim Sci. (2012) 92:227–59. doi: 10.4141/cjas2012-045

[15] Harris CM Madliger CL Love OP. Temporal overlap and repeatability of feather corticosterone levels: practical considerations for use as a biomarker. Conservation. Physiology. (2016) 4:4. doi: 10.1093/conphys/cow051, PMID: 27933163 PMC5142047

[16] Harris CM Madliger CL Love OP. An evaluation of feather corticosterone as a biomarker of fitness and an ecologically relevant stressor during breeding in the wild. Oecologia. (2017) 183:987–96. doi: 10.1007/s00442-017-3836-1, PMID: 28214946

[17] Hendrickx JO De Moudt S Calus E Martinet W Guns P-JD Roth L. Serum corticosterone and insulin resistance as early biomarkers in the hAPP23 overexpressing mouse model of Alzheimer’s disease. Int J Mol Sci. (2021) 22:6656. doi: 10.3390/ijms22136656, PMID: 34206322 PMC8269119

[18] Elsey RM Joanen T McNease L Lance V. Growth rate and plasma corticosterone levels in juvenile alligators maintained at different stocking densities. J Exp Zool. (1990) 255:30–6. doi: 10.1002/jez.1402550106

[19] Lance VA. Life in the slow lane: hormones, stress, and the immune system in reptiles. Perspectives Comparative Endocrinol. (1994) 1994:529–34.

[20] Lance VA Elsey RM. Stress-induced suppression of testosterone secretion in male alligators. J Exp Zool. (1986) 239:241–6. doi: 10.1002/jez.1402390211, PMID: 3746233

[21] Morici LA Elsey RM Lance VA. Effects of long-term corticosterone implants on growth and immune function in juvenile alligators, *Alligator mississippiensis*. J Exp Zool. (1997) 279:156–62. doi: 10.1002/(SICI)1097-010X(19971001)279:2<156::AID-JEZ6>3.0.CO;2-N, PMID: 9293640

[22] Franklin CE Davis BM Peucker S Stephenson H Mayer R Whittier J. Comparison of stress induced by manual restraint and immobilisation in the estuarine crocodile, *Crocodylus porosus*. J Exp Zool A Comp Exp Biol. (2003) 298:86–92. doi: 10.1002/jez.a.10233, PMID: 12884270

[23] Isberg SR Shilton CM. Stress in farmed saltwater crocodiles (*Crocodylus porosus*): no difference between individually-and communally-housed animals. Springerplus. (2013) 2:1–6. doi: 10.1186/2193-1801-2-381 24010039 PMC3755803

[24] Augustine L Miller K Peters A Franklin AD Steinbeiser CM Brown JL. Impacts of the season and reproductive status on fecal reproductive and adrenocortical steroid metabolites in zoo Cuban crocodiles (*Crocodylus rhombifer*). Zoo Biol. (2020) 39:411–21. doi: 10.1002/zoo.21559, PMID: 32770706

[25] Ganswindt SB Myburgh JG Cameron EZ Ganswindt A. Non-invasive assessment of adrenocortical function in captive Nile crocodiles (*Crocodylus niloticus*) part a molecular & integrative physiology. Comp Biochem Physiol A Mol Integr Physio. (2014) 177:11–7. doi: 10.1016/j.cbpa.2014.07.013 25066028

[26] Beale DJ Nguyen TV Shah RM Bissett A Nahar A Smith M. Host-gut microbiome metabolic interactions in PFAS-impacted freshwater turtles (*Emydura macquarii macquarii*). Meta. (2022) 12:747. doi: 10.3390/metabo12080747, PMID: 36005619 PMC9415956

[27] Abdelrhman KFA Bacci G Mancusi C Mengoni A Serena F Ugolini A. A first insight into the gut microbiota of the sea turtle *Caretta caretta*. Front Microbiol. (2016) 7:1060. doi: 10.3389/fmicb.2016.01060, PMID: 27458451 PMC4935691

[28] National Health and Medical Research Council. Australian code for the care and use of animals for scientific purposes. Canberra: National Health and Medical Research Council (2013).

[29] Campbell DLM Hewitt L Lee C Timmerhues CA Small AH. Behaviours of farmed saltwater crocodiles (*Crocodylus porosus*) housed individually or in groups. Frontiers in veterinary. Science. (2024) 11:11. doi: 10.3389/fvets.2024.1394198, PMID: 39040820 PMC11261483

[30] Beale DJ Hillyer K Nilsson S Limpus D Bose U Broadbent JA. Bioaccumulation and metabolic response of PFAS mixtures in wild-caught freshwater turtles (*Emydura macquarii*macquarii) using omics-based ecosurveillance techniques. Sci Total Environ. (2022) 806:151264. doi: 10.1016/j.scitotenv.2021.151264, PMID: 34715216

[31] Sartain M. (2016) The Agilent metabolomics dynamic MRM database and method. Agilent Technologies Technical Overview, publication number 5991-6482EN.

[32] Gyawali P Karpe AV Hillyer KE Nguyen TV Hewitt J Beale DJ. A multi-platform metabolomics approach to identify possible biomarkers for human faecal contamination in Greenshell™ mussels (Perna canaliculus). Sci Total Environ. (2021) 771:145363. doi: 10.1016/j.scitotenv.2021.145363, PMID: 33736167

[33] Shah RM Hillyer KE Stephenson S Crosswell J Karpe AV Palombo EA. Functional analysis of pristine estuarine marine sediments. Sci Total Environ. (2021) 781:146526. doi: 10.1016/j.scitotenv.2021.146526, PMID: 33798899

[34] Beale DJ Shah R Karpe AV Hillyer KE McAuley AJ Au GG. Metabolic profiling from an asymptomatic ferret model of SARS-CoV-2 infection. Meta. (2021) 11:327. doi: 10.3390/metabo11050327, PMID: 34069591 PMC8160988

[35] Pang Z Lu Y Zhou G Hui F Xu L Viau C. MetaboAnalyst 6.0: towards a unified platform for metabolomics data processing, analysis and interpretation. Nucleic Acids Res. (2024) 52:W398–406. doi: 10.1093/nar/gkae253, PMID: 38587201 PMC11223798

[36] Yu G Xu C Wang X Ju F Fu J Ni Y. MetOrigin 2.0: advancing the discovery of microbial metabolites and their origins. iMeta. (2024) e246. doi: 10.1002/imt2.246 39742299 PMC11683456

[37] Green RE Braun EL Armstrong J Earl D Nguyen N Hickey G. Three crocodilian genomes reveal ancestral patterns of evolution among archosaurs. Science. (2014) 346:1254449. doi: 10.1126/science.1254449, PMID: 25504731 PMC4386873

[38] De Livera AM Olshansky M Speed TP. Statistical analysis of metabolomics data In: Roessner U Dias DA, editors. Metabolomics tools for natural product discovery: Methods and protocols. Totowa, NJ: Humana Press (2013). 291–307. 10.1007/978-1-62703-577-4_20 23963918

[39] Agrawal A Balcı H Hanspers K Coort SL Martens M Slenter DN. WikiPathways 2024: next generation pathway database. Nucleic Acids Res. (2023) 52:D679–89. doi: 10.1093/nar/gkad960, PMID: 37941138 PMC10767877

[40] Caspi R Billington R Keseler IM Kothari A Krummenacker M Midford PE. The MetaCyc database of metabolic pathways and enzymes – a 2019 update. Nucleic Acids Res. (2020) 48:D445–d453. doi: 10.1093/nar/gkz862, PMID: 31586394 PMC6943030

[41] Karp PD Billington R Caspi R Fulcher CA Latendresse M Kothari A. The BioCyc collection of microbial genomes and metabolic pathways. Brief Bioinform. (2019) 20:1085–93. doi: 10.1093/bib/bbx085, PMID: 29447345 PMC6781571

[42] Lane D. (1991). 16S/23S rRNA sequencing. Nucleic acid techniques in bacterial systematics.

[43] Lane DJ Pace B Olsen GJ Stahl DA Sogin ML Pace NR. Rapid determination of 16S ribosomal RNA sequences for phylogenetic analyses. Proc Natl Acad Sci. (1985) 82:6955–9. doi: 10.1073/pnas.82.20.6955, PMID: 2413450 PMC391288

[44] Magoč T Salzberg SL. FLASH: fast length adjustment of short reads to improve genome assemblies. Bioinformatics. (2011) 27:2957–63. doi: 10.1093/bioinformatics/btr507, PMID: 21903629 PMC3198573

[45] Martin M. Cutadapt removes adapter sequences from high-throughput sequencing reads. EMBnet J. (2011) 17:3. doi: 10.14806/ej.17.1.200

[46] Edgar RC. Search and clustering orders of magnitude faster than BLAST. Bioinformatics. (2010) 26:2460–1. doi: 10.1093/bioinformatics/btq461, PMID: 20709691

[47] Glöckner FO Yilmaz P Quast C Gerken J Beccati A Ciuprina A. 25 years of serving the community with ribosomal RNA gene reference databases and tools. J Biotechnol. (2017) 261:169–76. doi: 10.1016/j.jbiotec.2017.06.1198, PMID: 28648396

[48] Quast C Pruesse E Yilmaz P Gerken J Schweer T Yarza P. The SILVA ribosomal RNA gene database project: improved data processing and web-based tools. Nucleic Acids Res. (2013) 41:D590–6. doi: 10.1093/nar/gks1219, PMID: 23193283 PMC3531112

[49] Yilmaz P Parfrey LW Yarza P Gerken J Pruesse E Quast C. The SILVA and "all-species living tree project (LTP)" taxonomic frameworks. Nucleic Acids Res. (2014) 42:D643–8. doi: 10.1093/nar/gkt1209, PMID: 24293649 PMC3965112

[50] Bolyen E Rideout JR Dillon MR Bokulich NA Abnet CC Al-Ghalith GA. Reproducible, interactive, scalable and extensible microbiome data science using QIIME 2. Nat Biotechnol. (2019) 37:852–7. doi: 10.1038/s41587-019-0209-9, PMID: 31341288 PMC7015180

[51] Davis NM Proctor DM Holmes SP Relman DA Callahan BJ. Simple statistical identification and removal of contaminant sequences in marker-gene and metagenomics data. Microbiome. (2018) 6:226. doi: 10.1186/s40168-018-0605-2, PMID: 30558668 PMC6298009

[52] Lloyd-Price J Arze C Ananthakrishnan AN Schirmer M Avila-Pacheco J Poon TW. Multi-omics of the gut microbial ecosystem in inflammatory bowel diseases. Nature. (2019) 569:655–62. doi: 10.1038/s41586-019-1237-9, PMID: 31142855 PMC6650278

[53] Hamilton LD Rellini AH Meston CM. Cortisol, sexual arousal, and affect in response to sexual stimuli. J Sex Med. (2008) 5:2111–8. doi: 10.1111/j.1743-6109.2008.00922.x, PMID: 18624961 PMC2703719

[54] Natelson BH Stokes PE Tapp WN. Plasma cortisol and appetitively motivated arousal in monkeys. Behav Neurosci. (1984) 98:925–9. doi: 10.1037/0735-7044.98.5.925, PMID: 6487422

[55] Riedemann T Patchev AV Cho K Almeida OFX. Corticosteroids: way upstream. Mol Brain. (2010) 3:2. doi: 10.1186/1756-6606-3-2, PMID: 20180948 PMC2841592

[56] Greaves RF Jevalikar G Hewitt JK Zacharin MR. A guide to understanding the steroid pathway: new insights and diagnostic implications. Clin Biochem. (2014) 47:5–15. doi: 10.1016/j.clinbiochem.2014.07.017, PMID: 25086367

[57] Liu C Chen J Liu B Liao WT Liu J Xu G. Activated corticosterone synthetic pathway is involved in poor responses to re-oxygenation after prolonged hypoxia. Int J Clin Exp Pathol. (2017) 10:8414–23. Available at: https://pmc.ncbi.nlm.nih.gov/articles/PMC6965453/pdf/ijcep0010-8414.pdf PMID: 31966693 PMC6965453

[58] Rowland NE Toth LA. Analytic and interpretational pitfalls to measuring fecal corticosterone metabolites in laboratory rats and mice. Comp Med. (2019) 69:337–49. doi: 10.30802/AALAS-CM-18-000119, PMID: 31578162 PMC6807723

[59] Gholiof M Adamson-De Luca E Wessels JM. The female reproductive tract microbiotas, inflammation, and gynecological conditions. Frontiers. Reprod Health. (2022) 4:4. doi: 10.3389/frph.2022.963752, PMID: 36303679 PMC9580710

[60] He S Li H Yu Z Zhang F Liang S Liu H. The gut microbiome and sex hormone-related diseases. Front Microbiol. (2021) 12:711137. doi: 10.3389/fmicb.2021.711137, PMID: 34650525 PMC8506209

[61] Neuman H Debelius JW Knight R Koren O. Microbial endocrinology: the interplay between the microbiota and the endocrine system. FEMS Microbiol Rev. (2015) 39:509–21. doi: 10.1093/femsre/fuu010, PMID: 25701044

[62] Beale DJ Sinclair GM Shah R Paten AM Kumar A Long SM. A review of omics-based PFAS exposure studies reveals common biochemical response pathways. Sci Total Environ. (2022) 845:157255. doi: 10.1016/j.scitotenv.2022.157255, PMID: 35817100

[63] National Center for Biotechnology Information. (2022). PubChem compound summary for CID 5312860, (R)-2-Hydroxycaprylic acid. Available at: https://pubchem.ncbi.nlm.nih.gov/compound/R_-2-Hydroxycaprylic-acid. (Accessed January 22, 2025).

[64] Wang B Sun Z Tu Y Si B Liu Y Yang L. Untargeted metabolomic investigate milk and ruminal fluid of Holstein cows supplemented with *Perilla frutescens* leaf. Food Res Int. (2021) 140:110017. doi: 10.1016/j.foodres.2020.110017, PMID: 33648248

[65] Jun H-I Kim B-T Song G-S Kim Y-S. Structural characterization of phenolic antioxidants from purple perilla (*Perilla frutescens* var. acuta) leaves. Food Chem. (2014) 148:367–72. doi: 10.1016/j.foodchem.2013.10.028, PMID: 24262570

[66] Sun Z Yu Z Wang B. *Perilla frutescens* leaf alters the rumen microbial community of lactating dairy cows. Microorganisms. (2019) 7:562. doi: 10.3390/microorganisms7110562 31766265 PMC6921060

[67] Yu H Qiu J-F Ma L-J Hu Y-J Li P Wan J-B. Phytochemical and phytopharmacological review of *Perilla frutescens* L.(Labiatae), a traditional edible-medicinal herb in China. Food Chem Toxicol. (2017) 108:375–91. doi: 10.1016/j.fct.2016.11.023, PMID: 27890564

[68] Wei M Zhao X Wang P Song X Hu J Zhong K. Novel metabolic biomarker for early detection and prognosis to the patients with gastric cardia Adnocarcinoma. Cancer Med. (2024) 13:e7015. doi: 10.1002/cam4.7015 38491808 PMC10943274

[69] Snider SR Kuchel O. Dopamine: an important Neurohormone of the Sympathoadrenal system. Significance of increased peripheral dopamine release for the human stress response and hypertension*. Endocr Rev. (1983) 4:291–309. doi: 10.1210/edrv-4-3-291, PMID: 6354703

[70] Berke JD. What does dopamine mean? Nat Neurosci. (2018) 21:787–93. doi: 10.1038/s41593-018-0152-y, PMID: 29760524 PMC6358212

[71] Cabib S Puglisi-Allegra S. The mesoaccumbens dopamine in coping with stress. Neurosci Biobehav Rev. (2012) 36:79–89. doi: 10.1016/j.neubiorev.2011.04.012, PMID: 21565217

[72] Barros RCH Branco LGS Cárnio EC. Evidence for thermoregulation by dopamine D1 and D2 receptors in the anteroventral preoptic region during normoxia and hypoxia. Brain Res. (2004) 1030:165–71. doi: 10.1016/j.brainres.2004.10.003, PMID: 15571666

[73] Lee TF Mora F Myers RD. Dopamine and thermoregulation: an evaluation with special reference to dopaminergic pathways. Neurosci Biobehav Rev. (1985) 9:589–98. doi: 10.1016/0149-7634(85)90005-3, PMID: 3001601

[74] Roelands B Meeusen R. Caffeine, dopamine and thermoregulation. Eur J Appl Physiol. (2012) 112:1979–80. doi: 10.1007/s00421-011-2127-5, PMID: 21874329

[75] Hepsomali P Groeger JA Nishihira J Scholey A. Effects of oral gamma-aminobutyric acid (GABA) administration on stress and sleep in humans: a systematic review. Front Neurosci. (2020) 14:923. doi: 10.3389/fnins.2020.00923, PMID: 33041752 PMC7527439

[76] Boonburapong B Laloknam S Incharoensakdi A. Accumulation of gamma-aminobutyric acid in the halotolerant cyanobacterium Aphanothece halophytica under salt and acid stress. J Appl Phycol. (2016) 28:141–8. doi: 10.1007/s10811-015-0523-7

[77] Kinnersley AM Turano FJ. Gamma aminobutyric acid (GABA) and plant responses to stress. Crit Rev Plant Sci. (2000) 19:479–509. doi: 10.1080/07352680091139277

[78] Hassel B Dahlberg D Mariussen E Goverud IL Antal EA Tønjum T. Brain infection with *Staphylococcus aureus* leads to high extracellular levels of glutamate, aspartate, γ-aminobutyric acid, and zinc. J Neurosci Res. (2014) 92:1792–800. doi: 10.1002/jnr.23444, PMID: 25043715

[79] Neal MJ Cunningham JR Shah MA Yazulla S. Immunocytochemical evidence that vigabatrin in rats causes GABA accumulation in glial cells of the retina. Neurosci Lett. (1989) 98:29–32. doi: 10.1016/0304-3940(89)90368-6, PMID: 2710396

[80] Connolly GP Duley JA. Uridine and its nucleotides: biological actions, therapeutic potentials. Trends Pharmacol Sci. (1999) 20:218–25. doi: 10.1016/S0165-6147(99)01298-5, PMID: 10354618

[81] Dobolyi A Juhász G Kovács Z Kardos J. Uridine function in the central nervous system. Curr Top Med Chem. (2011) 11:1058–67. doi: 10.2174/156802611795347618, PMID: 21401495

[82] Yamamoto T. 51 – relationship between exercise and beer ingestion in regard to metabolism In: Preedy VR, editor. Beer in health and disease prevention. San Diego: Academic Press (2009). 513–22.

[83] Yamamoto T Moriwaki Y Takahashi S Tsutsumi Z Yamakita J-i Higashino K. Effect of muscular exercise on the concentration of uridine and purine bases in plasma—adenosine triphosphate consumption—induced pyrimidine degradation. Metabolism. (1997) 46:1339–42. doi: 10.1016/S0026-0495(97)90241-9, PMID: 9361696

[84] Yamamoto T Moriwaki Y Takahashi S Tsutsumi Z Yamakita J-i Nakano T. Xylitol-induced increase in the plasma concentration and urinary excretion of uridine and purine bases. Metabolism. (1998) 47:739–43. doi: 10.1016/S0026-0495(98)90039-7, PMID: 9627375

[85] Yoshimi N Futamura T Kakumoto K Salehi AM Sellgren CM Holmén-Larsson J. Blood metabolomics analysis identifies abnormalities in the citric acid cycle, urea cycle, and amino acid metabolism in bipolar disorder. BBA Clin. (2016) 5:151–8. doi: 10.1016/j.bbacli.2016.03.008, PMID: 27114925 PMC4832124

[86] Li G Zhang X Qian H Liu M Zhao G Xu A. Gas chromatography-mass spectrometry based midgut metabolomics reveals the metabolic perturbations under NaF stress in *Bombyx mori*. Insects. (2020) 11:17. doi: 10.3390/insects11010017, PMID: 31878123 PMC7023488

[87] Foster ZSL Sharpton TJ Grünwald NJ. Metacoder: an R package for visualization and manipulation of community taxonomic diversity data. PLoS Comput Biol. (2017) 13:e1005404. doi: 10.1371/journal.pcbi.1005404, PMID: 28222096 PMC5340466

[88] Kaakoush NO. Insights into the role of Erysipelotrichaceae in the human host. Front Cell Infect Microbiol. (2015) 5:5. doi: 10.3389/fcimb.2015.00084, PMID: 26636046 PMC4653637

[89] Wu J Liu M Zhou M Wu L Yang H Huang L. Isolation and genomic characterization of five novel strains of Erysipelotrichaceae from commercial pigs. BMC Microbiol. (2021) 21:125. doi: 10.1186/s12866-021-02193-3, PMID: 33888068 PMC8063399

[90] Ali Q Ma S Farooq U Liu B Wang Z Sun H. Chronological dynamics of the gut microbiome in response to the pasture grazing system in geese. Microbiol Spectrum. (2024) 12:e04188–23. doi: 10.1128/spectrum.04188-23, PMID: 39189756 PMC11448393

[91] Neely WJ Martins RA Mendonça da Silva CM Ferreira da Silva T Fleck LE Whetstone RD. Linking microbiome and stress hormone responses in wild tropical treefrogs across continuous and fragmented forests. Communications Biology. (2023) 6:1261. doi: 10.1038/s42003-023-05600-9, PMID: 38087051 PMC10716138

[92] Sargsian S Mondragón-Palomino O Lejeune A Ercelen D Jin W-B Varghese A. Functional characterization of helminth-associated Clostridiales reveals covariates of Treg differentiation. Microbiome. (2024) 12:86. doi: 10.1186/s40168-024-01793-1, PMID: 38730492 PMC11084060

[93] Lopetuso LR Scaldaferri F Petito V Gasbarrini A. Commensal Clostridia: leading players in the maintenance of gut homeostasis. Gut Pathogens. (2013) 5:23. doi: 10.1186/1757-4749-5-23, PMID: 23941657 PMC3751348

[94] Stefanaki C Mastorakos G Chrousos GP. Gut microbiome and mental stress-related disorders: the interplay of classic and microbial endocrinology In: Gazouli M Theodoropoulos G, editors. Gut microbiome-related diseases and therapies. Cham: Springer International Publishing (2021). 229–42.

[95] Ringseis R Eder K. Heat stress in pigs and broilers: role of gut dysbiosis in the impairment of the gut-liver axis and restoration of these effects by probiotics, prebiotics and synbiotics. J Anim Sci Biotechnol. (2022) 13:126. doi: 10.1186/s40104-022-00783-3, PMID: 36397130 PMC9673442

[96] Prentki M Madiraju SRM. Glycerolipid metabolism and signaling in health and disease. Endocr Rev. (2008) 29:647–76. doi: 10.1210/er.2008-0007, PMID: 18606873

[97] Boros M Keppler F. Methane production and bioactivity-a link to Oxido-reductive stress. Front Physiol. (2019) 10:1244. doi: 10.3389/fphys.2019.01244, PMID: 31611816 PMC6776796

[98] Parthasarathy A Cross PJ Dobson RCJ Adams LE Savka MA Hudson AO. A three-ring Circus: metabolism of the three Proteogenic aromatic amino acids and their role in the health of plants and animals. Front Mol Biosci. (2018) 5:5. doi: 10.3389/fmolb.2018.00029, PMID: 29682508 PMC5897657

[99] Dhir S Tarasenko M Napoli E Giulivi C. Neurological, psychiatric, and biochemical aspects of thiamine deficiency in children and adults. Front Psych. (2019) 10:10. doi: 10.3389/fpsyt.2019.00207, PMID: 31019473 PMC6459027

[100] Huening KA Groves JT Wildenthal JA Tabita FR North JA. Escherichia colipossessing the dihydroxyacetone phosphate shunt utilize 5′-deoxynucleosides for growth. Microbiol Spectrum. (2024) 12:e03086–23. doi: 10.1128/spectrum.03086-23, PMID: 38441472 PMC10986504

[101] Lee JS Wang RX Alexeev EE Lanis JM Battista KD Glover LE. Hypoxanthine is a checkpoint stress metabolite in colonic epithelial energy modulation and barrier function. J Biol Chem. (2018) 293:6039–51. doi: 10.1074/jbc.RA117.000269, PMID: 29487135 PMC5912467

[102] Sainz F Jesús Torija M Matsutani M Kataoka N Yakushi T Matsushita K. Determination of dehydrogenase activities involved in D-glucose oxidation in Gluconobacter and Acetobacter strains. Front Microbiol. (2016) 7:1358. doi: 10.3389/fmicb.2016.01358, PMID: 27625643 PMC5003925

[103] Wakita Y Shimomura Y Kitada Y Yamamoto H Ohashi Y Matsumoto M. Taxonomic classification for microbiome analysis, which correlates well with the metabolite milieu of the gut. BMC Microbiol. (2018) 18:188. doi: 10.1186/s12866-018-1311-8, PMID: 30445918 PMC6240276

